# Body Temperature Differences Between Green And Brown Grasshoppers Do Not Result From Thermal Physiology or Thermal Preferences

**DOI:** 10.1002/ece3.71104

**Published:** 2025-03-11

**Authors:** Lilian Cabon, Holger Schielzeth

**Affiliations:** ^1^ Population Ecology Group, Institute of Ecology and Evolution Friedrich Schiller University Jena Jena Germany

**Keywords:** Acrididae, body mass, green–brown polymorphism, Orthoptera, preferred temperature, thermal physiology

## Abstract

Colour polymorphisms correspond to the co‐occurrence of several distinct colour morphs that vary in hue and/or brightness, independently of sex, age or any other state‐dependent modifiers. Colour morphs could represent different life‐history strategies, maximising their fitness locally in the trait space through trade‐offs between all their biological functions. This mechanism could play a role in the maintenance of the green–brown polymorphism in Orthoptera. Grasshoppers are characterised by a widespread green–brown polymorphism and continuous variability in brightness within colour morphs. It has previously been found that brown individuals are warmer in the field than green conspecifics, but it is unclear if these differences are related to thermal physiology and/or thermal preferences. Therefore, we experimentally tested the thermal physiology and thermal preferences of three green–brown polymorphic species of acridid grasshoppers. We found no differences between green and brown grasshoppers, either in heat‐up and equilibrium temperature patterns or in temperature preferences. Nor did we find support that the brightness variation is involved in the thermal physiology of these species. Instead, we show that body mass shapes the thermal physiology, with heavier individuals heating more slowly, and that males heated up faster and reached higher equilibrium temperatures than females. As females are heavier than males, the sex differences might be largely explained by size differences. Our results suggest that neither the thermal physiology nor the thermal preferences explain temperature differences in the field. However, green and brown individuals might still select different microhabitats in the field, which may indirectly lead to differences in body temperature. The persistence of the green–brown polymorphism may result from other mechanisms such as niche partitioning via microhabitat choice, mating preferences or frequency‐dependent apostatic selection.

## Graphical abstract

Colour morphs could represent different life‐history strategies, maximising their fitness locally in the trait space through trade‐offs between different biological functions. This mechanism could play a role in the maintenance of the green–brown polymorphism in Orthoptera. It has previously been found that brown individuals are warmer in the field than green conspecifics. These differences could result from morph‐specific thermal physiology and/or thermal preferences, for which we did not find support. Alternatively, the persistence of the green–brown polymorphism may result from mechanisms such as niche partitioning via microhabitat choice, mating preferences or frequency‐dependent apostatic selection.

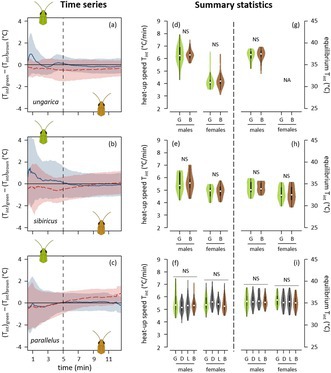

## 1 Introduction

Colour polymorphisms represent a particularly striking case of intraspecific diversity. They describe the co‐occurrence of distinct colour morphs independently of sex, age or any other state‐dependent modifiers (McKinnon and Pierotti 2010). Many colour polymorphisms are in a transient state and ultimately converge towards a monomorphic state either by natural selection or by genetic drift (Forsman et al. 2008; Moran 1992). Other colour polymorphisms seem to be balanced and are thus apparently maintained by balancing selection. The most potent process in balancing selection is negative frequency‐dependent selection (McKinnon and Pierotti 2010). However, any processes that equalise fitness between colour morphs can contribute to the maintenance of a polymorphism.

There is thus an interest in uncovering trade‐offs across different contexts. If one colour morph has an advantage in a particular context and a disadvantage in another, while it is reversed for the alternative colour morphs, the net fitness can be equal (Sheftel et al. 2018). Then, if there is intraspecific competition for microniches and the frequency of a morph increases, this morph will face a selective disadvantage by increased competition. This might lead to negative frequency‐dependent selection on alternative morphs stabilising the polymorphism. One benefit in many colour polymorphic prey species is crypsis (Franks and Oxford 2011; Endler 1988). However, crypsis may trade off with thermoecological processes, particularly in ectothermic organisms, such that some morphs are more cryptic, while other morphs can maintain high temperatures for longer, giving them larger time windows to feed, choose a mate or escape predators (Forsman 1999a). We here address the morph‐specific thermoecology in colour polymorphic grasshoppers.

One of the most prevalent colour polymorphisms is the green–brown polymorphism in Orthoptera. This polymorphism is found in about 30% of European orthopterans and 45% of East African acridid species (Schielzeth 2021; Rowell 1972), and the transspecies nature of the polymorphism suggests that it is maintained by balancing selection (Schielzeth 2021). There is also more subtle variation in body colouration in Orthoptera. In many species, populations are not only dimorphic for the presence or absence of green but also feature up to four colour morphs that vary in the distribution of green colouration. Furthermore, as in most organisms, there is also continuous variability in brightness within and between colour morphs, which might also affect the thermoecological properties. Interestingly, green and brown grasshoppers were found to differ in body temperature in the field (Cheng et al. 2022; Köhler and Schielzeth 2020). This pattern could be explained by morph‐specific thermal physiology and/or by morph‐specific behaviour such as behavioural thermoregulation. We here aim to explore the two alternatives.

Colour morph‐specific thermal physiology can result from the physical properties of colours that differ in the absorption of radiation, resulting in differential heat gain (Yu et al. 2017; Yurumezoglu et al. 2015). Indeed, dark morphs were found to have greater excess temperatures than light morphs in insects (Forsman 1997; Stewart and Dixon 1989; Brakefield and Willmer 1985). This has given rise to the thermal melanism hypothesis, according to which darker individuals should heat faster and reach higher equilibrium temperatures than equally sized brighter individuals (Clusella‐Trullas et al. 2008; de Jong et al. 1996; Watt 1968). The thermal melanism hypothesis has found empirical support both interspecifically (Storniolo et al. 2024; Zeuss et al. 2014; Clusella‐Trullas et al. 2008; Rapoport 1969) and to some extent intraspecifically (Britton and Davidowitz 2023; Zverev et al. 2018; Forsman 1997; de Jong et al. 1996; Brakefield and Willmer 1985; Watt 1968). So far, most of the studies have focused on discrete melanic polymorphisms (Clusella‐Trullas et al. 2007; Forsman 1997; Brakefield and Willmer 1985). The thermal physiology consequences for both intraspecific continuous colour variation and non‐melanic colour polymorphisms remain poorly explored.

In addition to colour itself, behavioural correlates of colour morphs may also affect thermoecology. Morph‐specific behavioural thermoregulation includes adopting specific postures such as sun‐bathing and/or selecting microhabitats based on thermal requirements. Sun‐bathing by flanking affects body temperature in ectotherms by controlling the exposure of specific body parts to radiation, allowing them to elevate body temperature well above ambient temperatures (Kingsolver 1987; Whitman 1987; Digby 1955). Thermoregulatory microhabitat choice by ectotherms can be done by choosing areas based on sun exposure and/or local temperature (Ahnesjö and Forsman 2006; Bundey et al. 2003). Behavioural thermoregulation might incur various costs, such as reduced time for feeding and mate search, or it might affect the risk of predation when individuals have to seek exposed locations for heat‐up. Behavioural thermoregulation can interact with body colouration (Nielsen et al. 2018; Kingsolver 1987), with brighter individuals potentially investing more in behavioural thermoregulation than darker individuals to counteract the effect of their lower thermal capacity (Stuart‐Fox et al. 2017). This could translate into morph‐specific thermal preferences.

We studied the thermal physiology and thermal preferences of green–brown polymorphic grasshoppers as non‐mutually exclusive causal explanations for morph‐specific body temperature differences measured in the field (Cheng et al. 2022; Köhler and Schielzeth 2020). Heat‐up patterns, equilibrium temperatures and preferred temperatures were tested in three green–brown polymorphic orthopteran species: the slant‐faced grasshopper *Acrida ungarica*, the club‐legged grasshopper *Gomphocerus sibiricus* and the meadow grasshopper *Pseudochorthippus parallelus* (all Caelifera, Acrididae). *Acrida ungarica* is a northern Mediterranean species inhabiting dry and warm grasslands, *Gomphocerus sibiricus* is an Alpine species found at altitudes between 1100 and 2900 m in Europe, and *Pseudochorthippus parallelus* is a more widespread species in Western and Central Europe inhabiting a large variety of temperate grasslands at altitudes between 0 and 2700 m (www.gbif.org; Giuliano 2022). Interestingly, *Acrida ungarica* is able to switch between green and brown when they moult, while the other two species (like most Gomphocerinae apparently) have simple genetic colour polymorphism determination and no plasticity to switch between colour morphs (Valverde and Schielzeth 2015; Winter et al. 2021).

Since brown individuals had higher body temperatures than green individuals in the field (Cheng et al. 2022; Köhler and Schielzeth 2020), we predict that brown individuals heat faster and reach higher equilibrium temperatures than green individuals. The thermal melanism hypothesis further predicts that darker individuals (within and between colour morphs) heat faster and reach higher equilibrium temperatures than brighter individuals. Alternatively, if thermal physiology does not induce colour morph‐specific body temperatures, field results are likely the outcome of thermal preferences. In that case, we predict that the brown morph prefers higher temperatures than the green morph. If thermal physiology otherwise does induce colour morph‐specific body temperatures, the predictions on thermal preferences are more complex. Here, the thermal preference effects can add to or partially cancel out the thermal physiology effects (brown prefers higher or lower temperatures, respectively) or even be absent when the thermal physiology effects alone are strong enough. Furthermore, assuming colouration, physiology and behaviour evolved in concert in our species, as suggested for groundhoppers (Forsman 2000, 1999a), we expect colour plastic individuals to have a greater optimal physiological range than non‐plastic individuals. We thus predict that any morph‐related differences in thermal preferences are less pronounced in colour‐changing species (slant‐faced grasshopper) compared to non‐plastic species (club‐legged and meadow grasshoppers).

## 2 Materials and Methods

### 2.1 Sampling

We studied the colour morphs of *Acrida ungarica*, *Pseudochorthippus parallelus* and *Gomphocerus sibiricus* (hereafter simply referred as to *ungarica*, *sibiricus* and *parallelus*, respectively). Individuals were collected in the field during the 2023 summer season (in mid to late June in east‐central Germany for *parallelus*; in mid‐July and mid‐august in northern Italy for *ungarica*; in mid‐July in the French Alps for *sibiricus*). *Ungarica* were collected as nymphs; *parallelus* and *sibiricus* were collected mostly as nymphs. The first sampling of *ungarica* led to an over‐representation of males because field sex ratios were highly unbalanced (17:2, males:females). We, therefore, oversampled females to compensate for missing individuals during the second sampling campaign.

**FIGURE 1:**
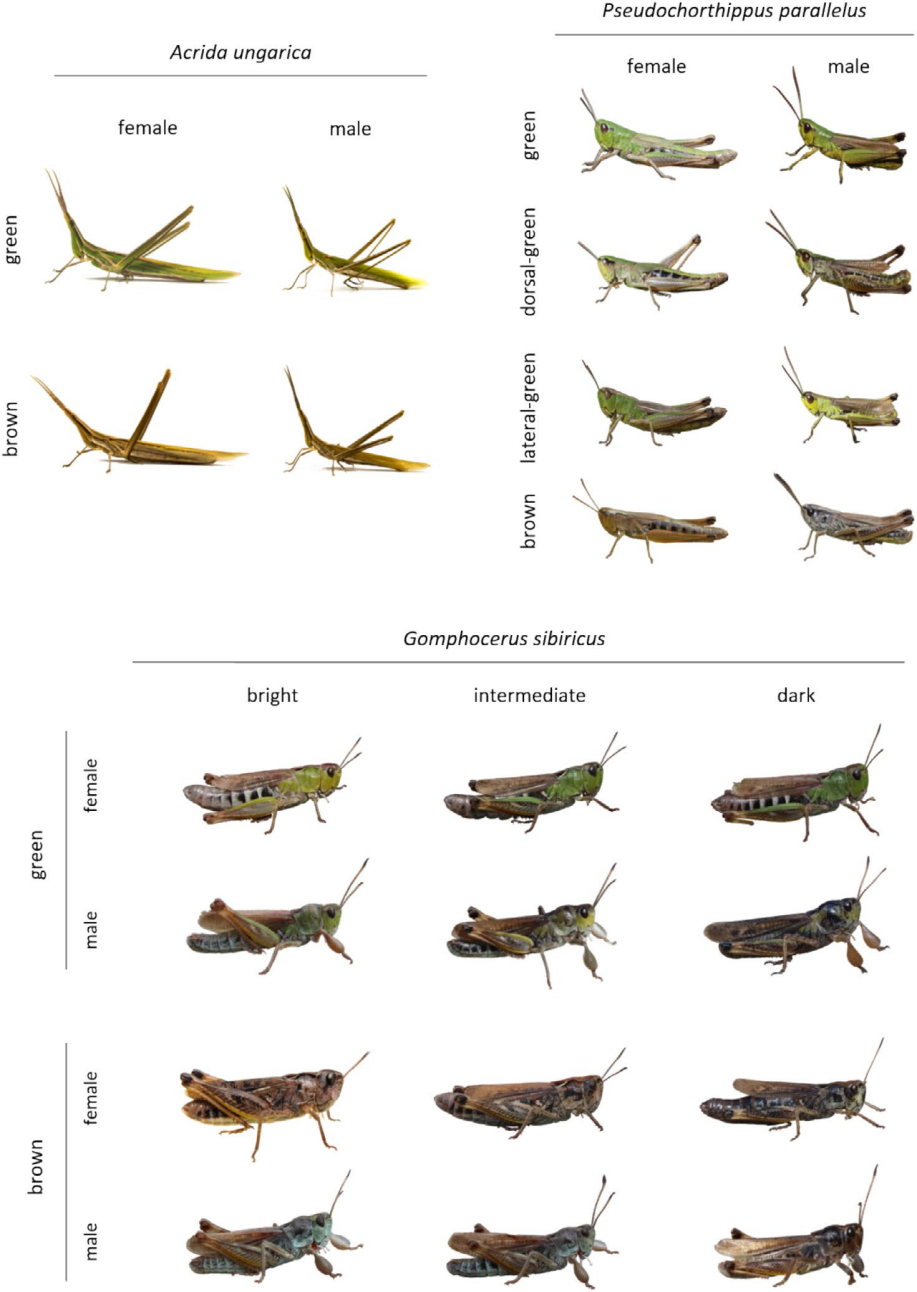
Colour variation in *Acrida ungarica*, *Pseudochorthippus parallelus* and *Gomphocerus sibiricus*. *Ungarica* and *sibiricus* present two distinct colour morphs, while *parallelus* have four, all characterised by the presence and distribution of the green colouration. The continuous brightness colour variation is exemplified in *sibiricus* only but exists in *ungarica* and *parallelus* as well. Pictures were shot under natural light and illustrate the overall patterns of interest.

All three species are green–brown polymorphic (Figure 1). *Ungarica* features two colour morphs: green and brown. *Sibiricus* presents green and brown individuals. Brown *sibiricus* can be subdivided into a plain‐brown morph and a pied morph. Although the difference between brown and pied is rather subtle and both are characterised by the lack of green (Schielzeth and Dieker 2020). Due to limited sample sizes of pied morphs and since field results—that motivated this study—pooled pied and plain‐brown (Köhler and Schielzeth 2020), we considered as the ‘brown’ morph both pied and plain‐brown individuals. *Parallelus* features four colour morphs characterised by dorsal‐lateral variation in the distribution of green. Individuals can be uniform green (laterally and dorsally green), lateral green (laterally green and dorsally brown), dorsal green (laterally brown and dorsally green) or uniform brown (laterally and dorsally brown). All species show continuous colour variation in brightness within their discrete colour morphs, resulting in significant inter‐individual differences. Body colours may range from light‐yellowish green (and brown) to rich dark green (and dark brown, sometimes almost blackish).

### 2.2 Housing & Weighing

Field‐caught grasshoppers were kept and raised to adulthood under controlled conditions in the lab (70% humidity, temperature around 20°C at night and around 35°C during the day). Grasshoppers were fed ad libitum with freshly cut grass blades, provided with a water tube for humidity and exposed to visible and UV lights (visible lights on from 6 am and 10 pm, and UV lights on from 8 am to 8 pm). Only adults with fixed colour morph were used for the experiments and, therefore, the colour morph of *ungarica* individuals remained unchanged throughout the experiments. Prior to the experiments, all individuals were weighed with a microbalance (XS105 Mettler Toledo, ±0.1 mg). Each individual was tested on the same day in both experimental set‐ups presented below (thermal preferences tested between 10 am and 1 pm, thermal physiology tested between 1 pm and 7 pm).

### 2.3 Thermal Preferences

We tested thermal preferences using thermal gradients. Each gradient set‐up was made of an 88 × 41.5 × 0.8 cm aluminium plate mounted on 22 cm high aluminium feet. The preferred body temperature of different acridid species is known to range from 22°C to 41°C (Harris et al. 2013; Springate and Thomas 2005; Blanford and Thomas 2000). The thermal amplitude of the gradients was set up to contain this range by bathing one foot in ice and the other in water heated to 60°C (using an immersion heater [Rommelsbacher TS 2003, 2000 W] and a thermostat [Schego thermostat TR2]). This yielded the runways to range from 10.0°C ± 1.1°C to 51.0°C ± 2.3°C (mean ± SD). Plexiglass dividers were mounted vertically on top to create four identical runways of 80 × 8 cm. The lower part of the Plexiglass dividers was coated with Fluon (PTFE) up to 12 cm height to prevent grasshoppers from climbing the walls. The gradients were covered with a thin plexiglass lid allowing the direct observation of the grasshoppers' positions without disturbance. The set‐up was lit with fluorescent tubes. Using four identical set‐ups, we were able to test 16 individuals in one session. All recording sessions took place between 10 am and 1 pm.

Before starting the experiment, the temperature of each runway was measured at 5 equally spaced positions (distance from cold edge: 0, 20, 40, 60 and 80 cm) using a handheld infrared thermometer (Farnell, dual focus infrared thermometer, ±0.1°C). We interpolated temperatures at intermediate positions by fitting a degree 3 polynomial with the *nls* function from R. Since the drift in temperatures for the duration of the experiment was relatively low (0.9°C ± 0.2°C, mean ± SD), we measured the temperature of the gradient only once at the beginning of the session. Individuals were then released in the middle of the runway (27.1°C ± 1.3°C, mean ± SD) and were free to move for an hour. Their positions were recorded every 10 min, leading to six measures per individual. Their positions were converted to temperatures for further analyses using the *nls* fits described above. When an individual was seen perching on the wall or the lid, it was gently directed towards the middle of the runway, and missing data were recorded for that reading (1.6% of all sightings).

On each gradient set‐up, we ensured that morphs and as much as possible sexes were balanced to minimise confounding effects (see *Thermal physiology* since we used the same groups of animals in both experiments).

### 2.4 Thermal Physiology

Adaptive variation in animal colouration, particularly in visible reflectance, represents a trade‐off between various competing functions, including camouflage and thermoregulation. Alternatively, adaptive variation in infrared reflectance is likely driven predominantly by thermoregulatory needs, as most animal visual systems are largely insensitive to near‐infrared wavelengths (Stuart‐Fox et al. 2017). Hence, we tested heat‐up patterns and equilibrium temperatures under a radiant infrared regime. To monitor internal body temperature, we inserted the tip of a K‐type thermocouple connected to a datalogger into the ventral side of the thorax of living grasshoppers piercing the intersegmental membrane between the thorax and the abdomen. The thorax was then glued (KIMTEC sekundenkleber speed 40, ethyl‐2‐cyanacrylat) onto a small pole of 7 mm in diameter. The pole was mounted onto a platform that held the thermocouple so that the set‐up stayed immobile throughout the experiment. Once glued, grasshoppers were placed in a freezer for 10 min to chill them to a temperature of 5.8°C ± 3.0°C (mean ± standard deviation, SD). The grasshoppers were then placed under an infrared lamp emitting near‐ and mid‐infrared radiation (Elstein HTS/1, 230 V, 400 W, wavelength range: 2–10 μm) placed 41 cm above the grasshoppers. The K‐type thermocouples recorded the internal temperature of the thorax every 5 s. At the same time, a thermal camera (HIKMICRO B20, ±2°C) positioned in front of the experimental set‐up at an angle of about 60° took pictures every 20 s. Using the spot function of the HIKMICRO Analyser software (v.1.3.1.5), we measured the external temperature of the thorax.

One experimental session comprised four grasshoppers. We ensured that morphs were balanced (2 greens and 2 browns for *ungarica* and *sibiricus*, and 1 individual of each 4 morphs for *parallelus*) within sessions and that sexes were also balanced as much as possible (due to unbalanced sex ratios in *ungarica*, 4 sessions were sex‐balanced while 15 were male‐only, and 13 were female‐only). The experiment lasted for 13.2 ± 0.7 min (mean ± SD), which allowed to reach equilibrium for most experimental sessions. For the analyses, we truncated the first 20 s because internal temperatures kept going down during this period in 24% of the individuals, likely due to temperature homogenisation via circulating haemolymph and were not representative of the heat‐up effect of the radiant lamp.

### 2.5 Brightness Measurements

Individuals were placed in the refrigerator overnight following the experiments. The next day, body colour was measured using a handheld spectrophotometer (Avantes, AvaSpec‐ULS2048x16; optic fibre: FCR‐7UVIR200‐2‐1.5X100, 1.5 mm diameter) coupled with a deuterium‐halogen light source (Avantes, AvaLight‐DH‐S). The device was calibrated with a commercial white standard (Avantes WS‐2) on each day. The AvaSoft 7 software (Avantes, v.7.8) captured the reflectance spectra configured with an integration time of 100 ms and an automatic averaging of five readings. Reflectance between 300 and 1000 nm was measured in a dark room, holding the probe at a 45° angle while gently touching the cuticle surface. Our device always displays a narrow peak in reflectance between 654 and 659 nm. This peak apparently represents an artefact of the device and was replaced by the average across reflectance at 650–654 nm and 659–664 nm.

Reflectance spectra were captured for the dorsal side and the right lateral lobe of the pronotum. Grasshopper body parts display complex colouration patterns (Figure 1). To account for this variability, we took five independent measurements of both body parts. For each individual, we averaged reflectance across these 10 measurements using the *aggspec* function from the *pavo* package (version 2.9.0, on R) (Maia et al. 2019). This led to a total of 432 reflectance spectra. We then derived the brightness value from each spectrum using the *summary.rspec* function from *pavo* by extracting the mean brightness. Mean brightness, simply referred to as brightness hereafter, corresponds to the mean relative reflectance over the entire spectral range, that is, 300–1000 nm here. We deem this measure of brightness to be physiologically relevant, but note that it is different from how animals perceive brightness, since perception depends on the specificities of the visual system.

### 2.6 Replication Statement

For a description of the replication design we used for each of our three predictors (colour morph, brightness and weight), see Table 1.

**TABLE 1:** Replication statement for our study on thermal physiology and thermal preferences of three green–brown polymorphic species of grasshoppers.

Scale of inference	Scale at which the factor of interest is applied	Number of replicates at the appropriate scale
Colour morphs	Individuals	*Acrida ungarica*: 30 replicates for each morph in females and 34 in males *Gomphocerus sibiricus*: 19 replicates for each morph in females and 13 in males *Pseudochorthippus parallelus*: 30 replicates for each morph in males and females
Individuals (brightness and weight effect)	Individuals	*Acrida ungarica*: 60 replicates in females and 68 in males *Gomphocerus sibiricus*: 38 replicates in females and 26 in males *Pseudochorthippus parallelus*: 120 replicates for each sex

*Note:* The dataset has been subdivided by species and by sex, leading to six subsets. Each species **×** sex subset acts as a replicate for the inference we are depicting. We describe the number of replicates for each of these six subsets.

### 2.7 Statistical Analyses

All descriptive statistics given in the results are presented as the mean ± standard deviation. All statistical analyses were performed on R version 4.2.2 (R Core Team 2023).

We analysed the three species and two sexes separately by subsetting species and sex (six subsets in total). Within each subset, we tested for morph differences as well as for the effects of brightness and body weight as predictors. Confidence intervals (CI) were estimated by non‐parametric bootstrapping with 1000 iterations. Bootstrap resamples were made out of complete time series of individuals that were resampled with replacement while constraining each resample to have the same sample size for each colour morph as in the original subset. For each bootstrap iteration, we calculated the statistic of interest—differences in means between morphs or Spearman's correlation coefficients between brightness (or weight) and the response variable (internal temperature, external temperature or substrate temperature). We derived the 95% CI from the distribution of the statistic of interest using the percentile method. Differences in means or correlation coefficients were treated as significant if the bootstrapped 95% CI did not contain zero. When testing the colour morph effect (e.g., mean_green_—mean_brown_), a CI above zero indicates that green individuals were hotter (when internal or external temperatures are tested) or that they were standing on hotter spots (when the substrate temperature is tested) than brown individuals. When testing the brightness (or weight) effect, a positive effect size means that brighter (or heavier) individuals were hotter or that they were standing on hotter spots than darker (or lighter) individuals.

We derived three summary statistics from the time series. From the thermal physiology time series, we derived the *heat‐up speed*, which represents the rate of temperature change between the 20th and 240th seconds of the experiment, and the *equilibrium temperature*, which averages the temperature reached by an individual between the 660th and 760th seconds of the experiment. Both of those statistics have been derived for the internal and external temperatures. From the thermal preferences time series, we derived the *preferred temperature*, which corresponds to the average temperature of the substrate where the individual was standing between the 30th and 60th minutes.

**FIGURE 2:**
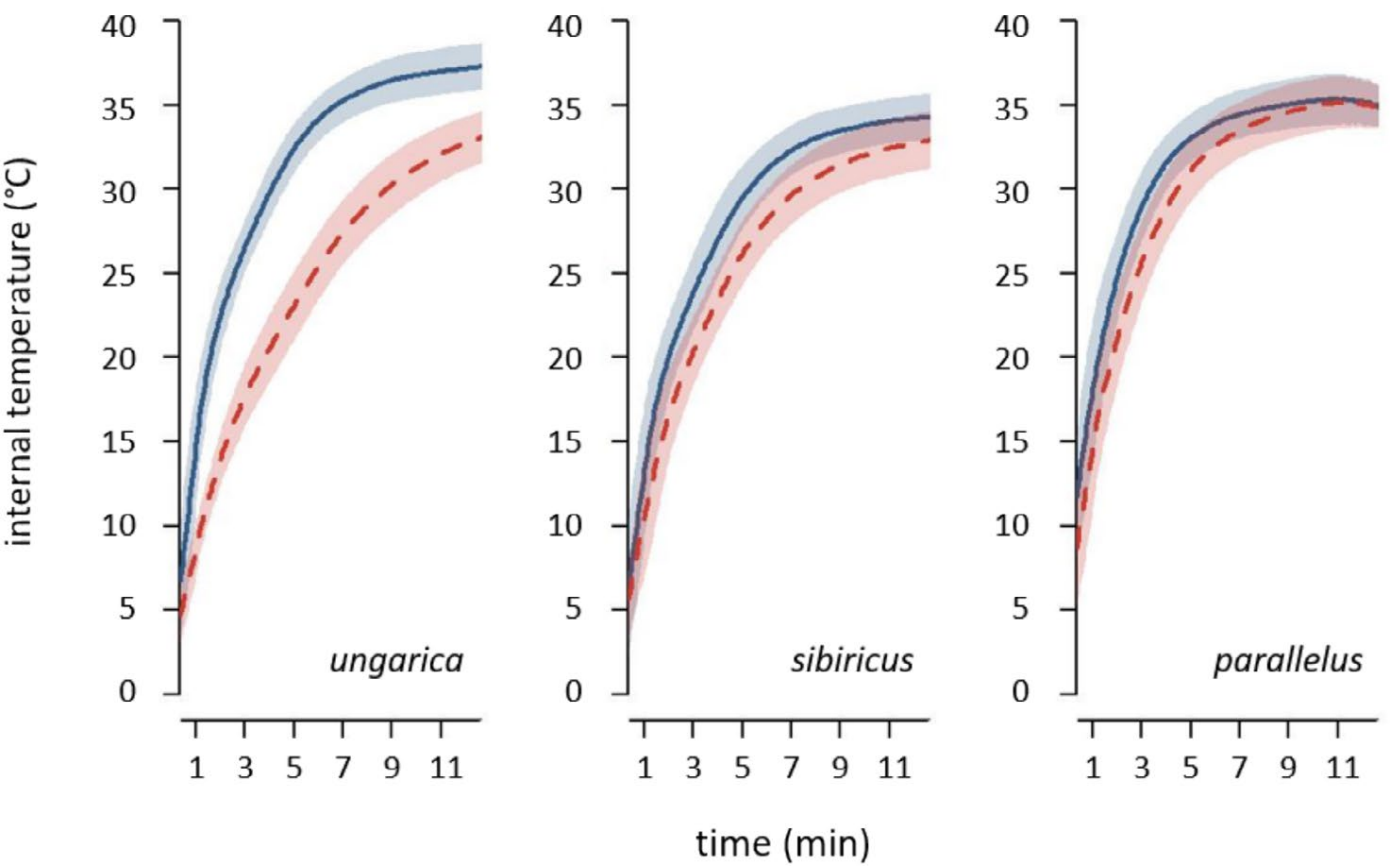
Internal temperature depending on time for both sexes (solid blue curves: Males, dashed red curves: Females) of *Acrida ungarica*, *Gomphocerus sibiricus* and *Pseudochorthippus parallelus* (from left to right, abbreviated respectively *ungarica*, *sibiricus* and *parallelus*). Curves represent the average heat‐up trajectories; ribbons represent the standard deviations.

Normality was checked from quantile–quantile plots. Homoscedasticity was checked using Fisher tests (two levels) or Bartlett tests (more than two levels). When heteroscedasticity was found, data were log‐transformed to meet homoscedasticity when possible (it only concerned the heat‐up speed for *parallelus* males tested against morph and for *sibiricus* tested against sex). The morph effect was tested using *t*‐tests (when normality and homoscedasticity were met), Welch tests (when normality only was met) and Mann–Whitney tests (when normality was not met) for *ungarica* and *sibiricus*, and using one‐way ANOVAs for *parallelus*. For the Kruskal–Wallis tests featuring a *p* ≤ 0.05, means were separated using pairwise Mann–Whitney tests. The brightness and weight effects were tested using Pearson's correlation tests when bivariate normality was met or Spearman's correlation tests otherwise. Confidence intervals (CI) were derived from correlation coefficients of 1000 bootstrap resamples (sampling with replacement while constraining the resample to have the same sample size as its original dataset). Correlation coefficients were treated as significant if the bootstrapped 95% CI did not contain zero. The sex effect on the summary statistics was tested using Mann–Whitney tests when normality was not met, and using *t* tests otherwise. Tests on equilibrium temperatures of *ungarica* females were not performed because no equilibrium was reached (Figure 2, Figure S1).

## 3 Results

We tested the heat‐up capacity and temperature preferences of 432 grasshoppers of three species: *Acrida ungarica* (60 females and 68 males), *Gomphocerus sibiricus* (38 females and 26 males) and *Pseudochorthippus parallelus* (120 females and 120 males). Body weight also differed across species (*χ* ^2^ = 160, df = 2, *p* < 0.001), with *ungarica* being about twice as heavy as *sibiricus* (*W* = 5144, *p* < 0.01; *ungarica*: 0.64 ± 0.49 g; *sibiricus*: 0.29 ± 0.10 g) and *sibiricus* being heavier than *parallelus* (*W* = 13,015, *p* < 0.001, *parallelus*: 0.16 ± 0.07 g).

The heat‐up pattern can be divided into two phases: the heat‐up phase and the equilibrium phase. The heat‐up phase is characterised by a fast increase in temperature over a short period of time at the beginning of the experiment (Figure 2, Figures S1 and S2a–f). For our study, the equilibrium phase is defined as a temperature plateau where the temperature remains stable through time or slightly increases towards a plateau. The heat‐up phase is thus denoted by the first 5 min of the experiment for all of our grasshoppers, except for *ungarica* females, which did not reach the equilibrium phase (Figure 2, Figure S1). The results reported below on the equilibrium phase thus exclude *ungarica* females.

### 3.1 Effects of the Discrete Colour Morphs

**FIGURE 3:**
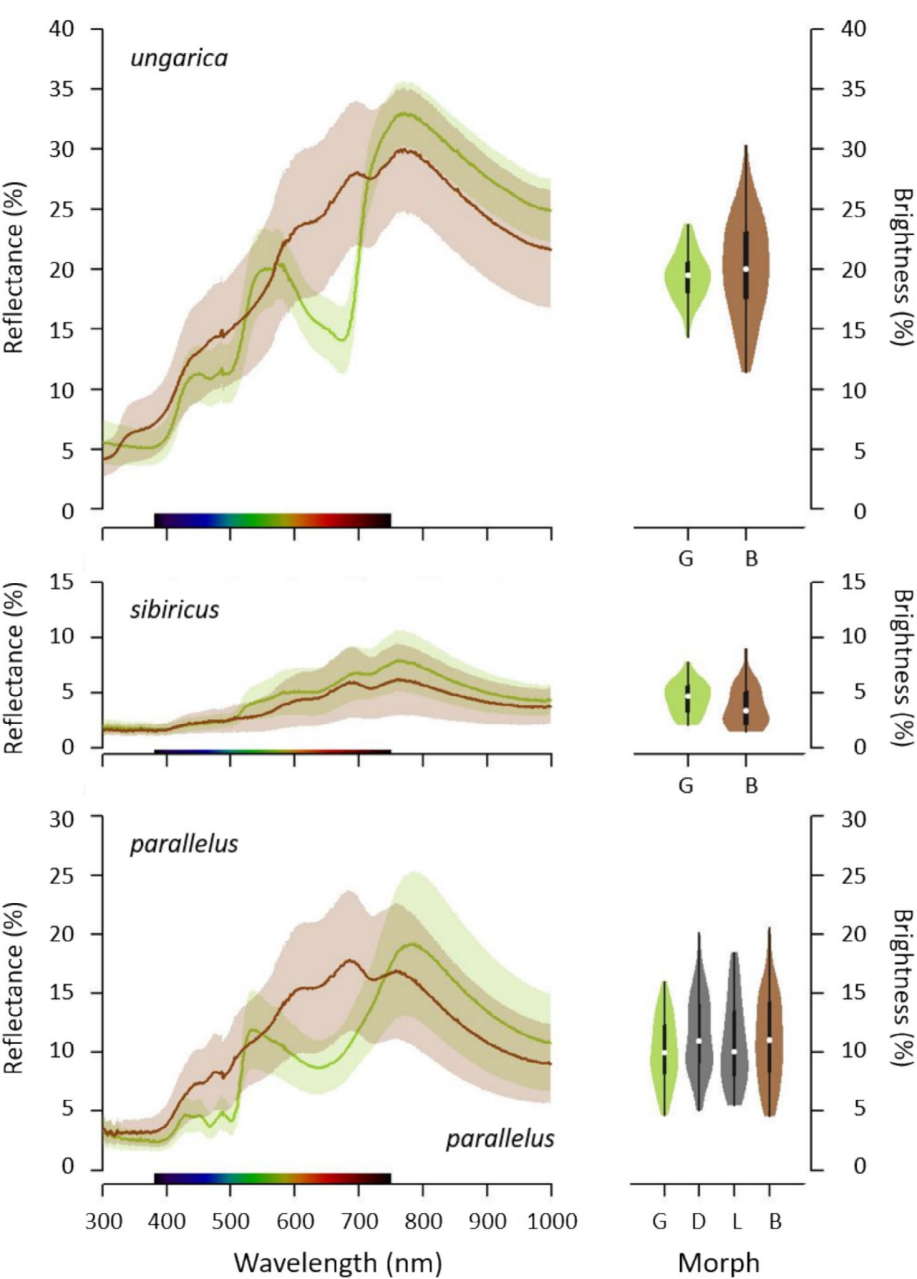
Average reflectance spectra (left) and mean brightness (right) of green–brown polymorphic grasshoppers for three different species: *Acrida ungarica*, *Gomphocerus sibiricus* and *Pseudochorthippus parallelus* (from top to bottom, abbreviated respectively *ungarica*, *sibiricus* and *parallelus*). The reflectance spectra were calculated with only fully green and fully brown individuals. Curves/points represent the mean; ribbons/arrows represent the standard deviations. Average reflectance of green and brown individuals is shown in green and brown, respectively. G, D, L and B mean green, dorsal‐green, lateral‐green and brown morph, respectively.

The brightness did not differ significantly among colour morphs (Figure 3; *ungarica*: *t* = −1.26, df = 92, *p* = 0.21; *sibiricus*: *W* = 658, *p* = 0.05; *parallelus*: *F* = 1.58, df = 3, *p* = 0.20). Hence, colour morphs were not confounded with brightness.

**FIGURE 4:**
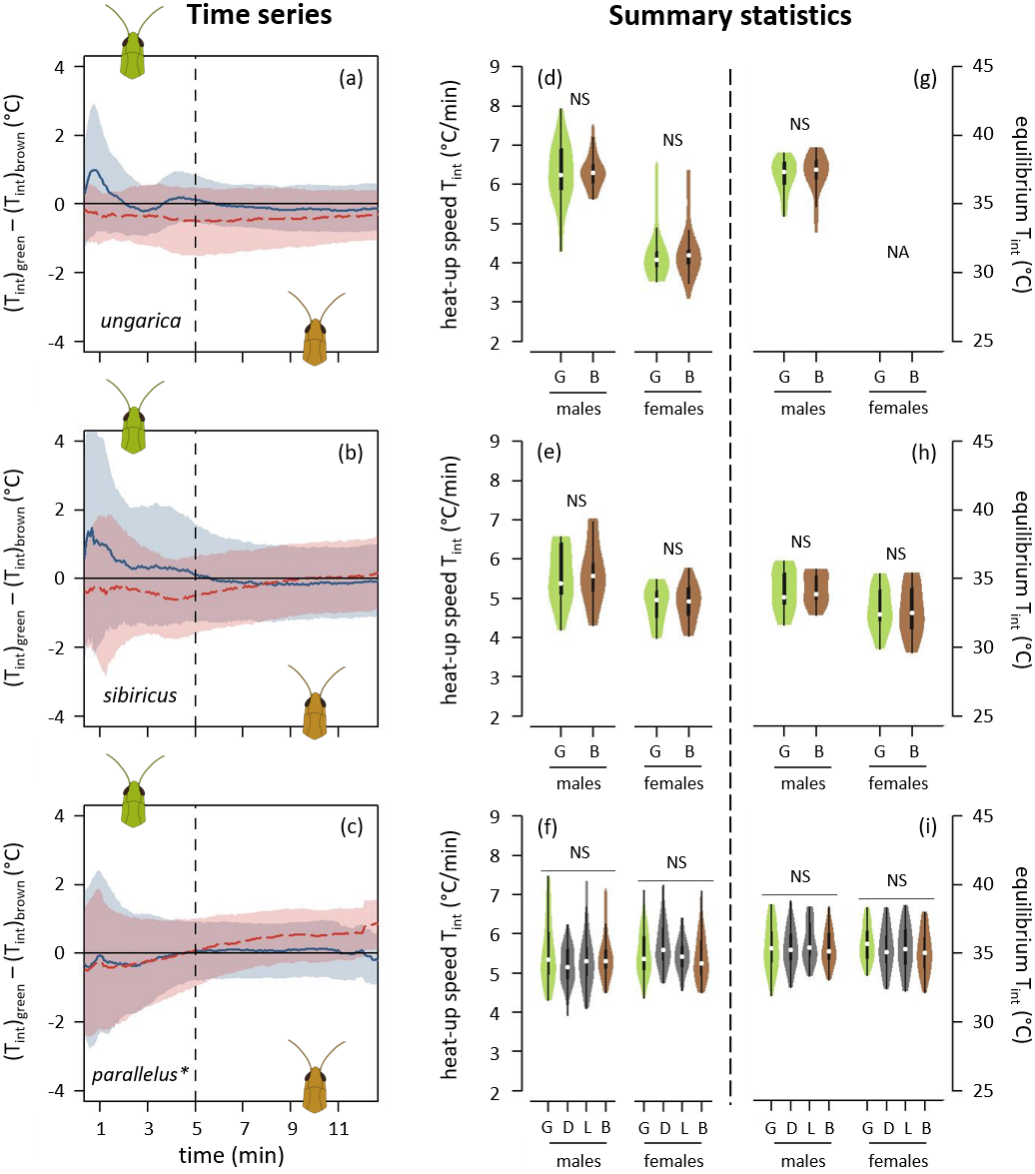
Thermal physiology depending on morphs for *Acrida ungarica*, *Gomphocerus sibiricus* and Pseudochorthippus parallelus* (from top to bottom, abbreviated respectively *ungarica*, *sibiricus* and *parallelus*). The dotted vertical lines distinguish between the heat‐up phase (left) and the equilibrium phase (right). (a–c) Pairwise differences in internal temperature (T_int_) between green and brown morphs depending on time (T_green_—T_brown_). Each species' dataset was split into males (solid blue curves) and females (dashed red curves). When a curve goes above the zero line, it indicates that the green morph was hotter than the brown morph. Curves represent average differences. Ribbons represent bootstrapped 95% confidence intervals. The dashed vertical lines separate the heat‐up phase (before) and the equilibrium phase (after). (d–f) Internal heat‐up speed. (g–i) Internal equilibrium temperature. (d–i) Violins represent densities of observations and their boxplots with medians indicated by a white dot. G, D, L and B stand for green, dorsal‐green, lateral‐green and brown morphs, and are shown in green, grey, grey and brown, respectively. NS means non‐significant. *Ungarica* females did not reach any equilibrium, and their equilibrium temperatures are, hence, not available (NA). *For the pairwise differences involving the two intermediate morphs (dorsal green and lateral green), see Figure S4a–e.

**FIGURE 5:**
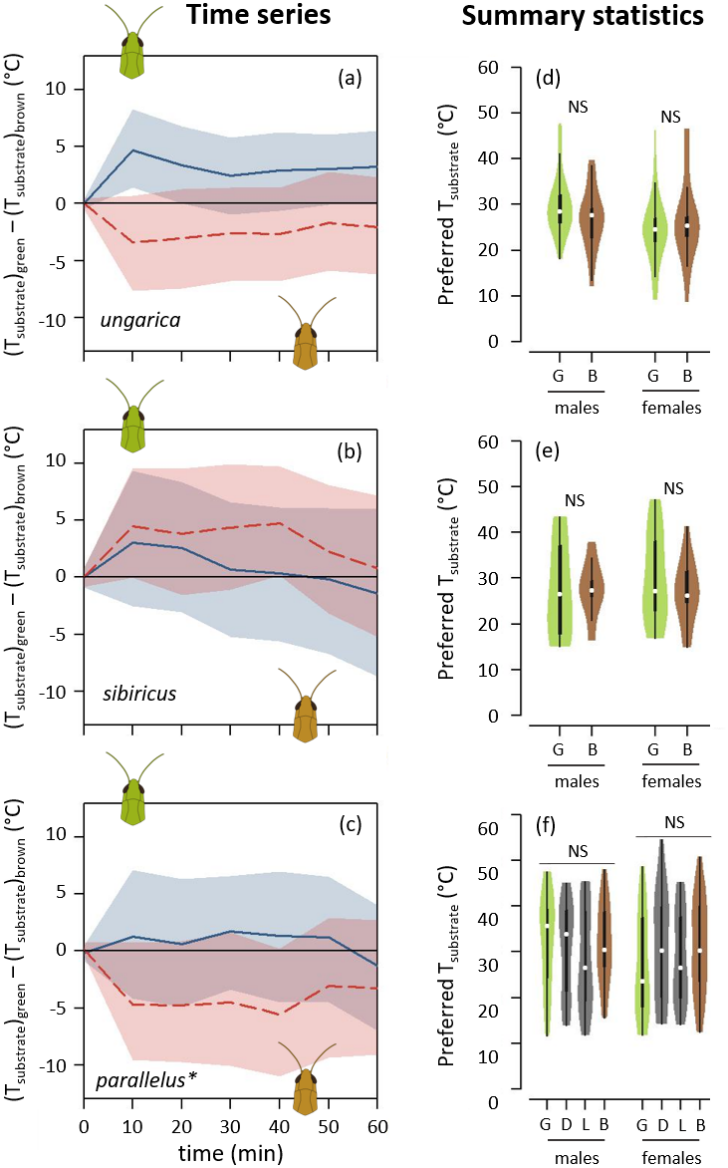
Thermal preferences depending on morphs for *Acrida ungarica*, *Gomphocerus sibiricus* and Pseudochorthippus parallelus* (from top to bottom, abbreviated respectively *ungarica*, *sibiricus* and *parallelus*). (a–c) Pairwise differences in substrate temperature (T_substrate_) between green and brown morphs depending on time (T_green_—T_brown_). Each species' dataset was split into males (solid blue curves) and females (dashed red curves). When a curve goes above the zero line, it indicates that the green morph was standing on hotter temperatures than the brown morph. See Figure 4 caption for details on graphical displays. (d–f) Preferred temperature. *For the pairwise differences involving the two intermediate morphs (dorsal green and lateral green), see Figure S4k–o. See Figure 4 caption for details on graphical displays.

Neither the heat‐up phase nor the equilibrium phase differed among the colour morphs in the two sexes across all species (Figure 4, Figures S3 and S4a–j). Likewise, different colour morphs had similar preferred temperatures (Figure 5, Figure S4k–o).

### 3.2 Effects of the Continuous Colour Variation

**FIGURE 6:**
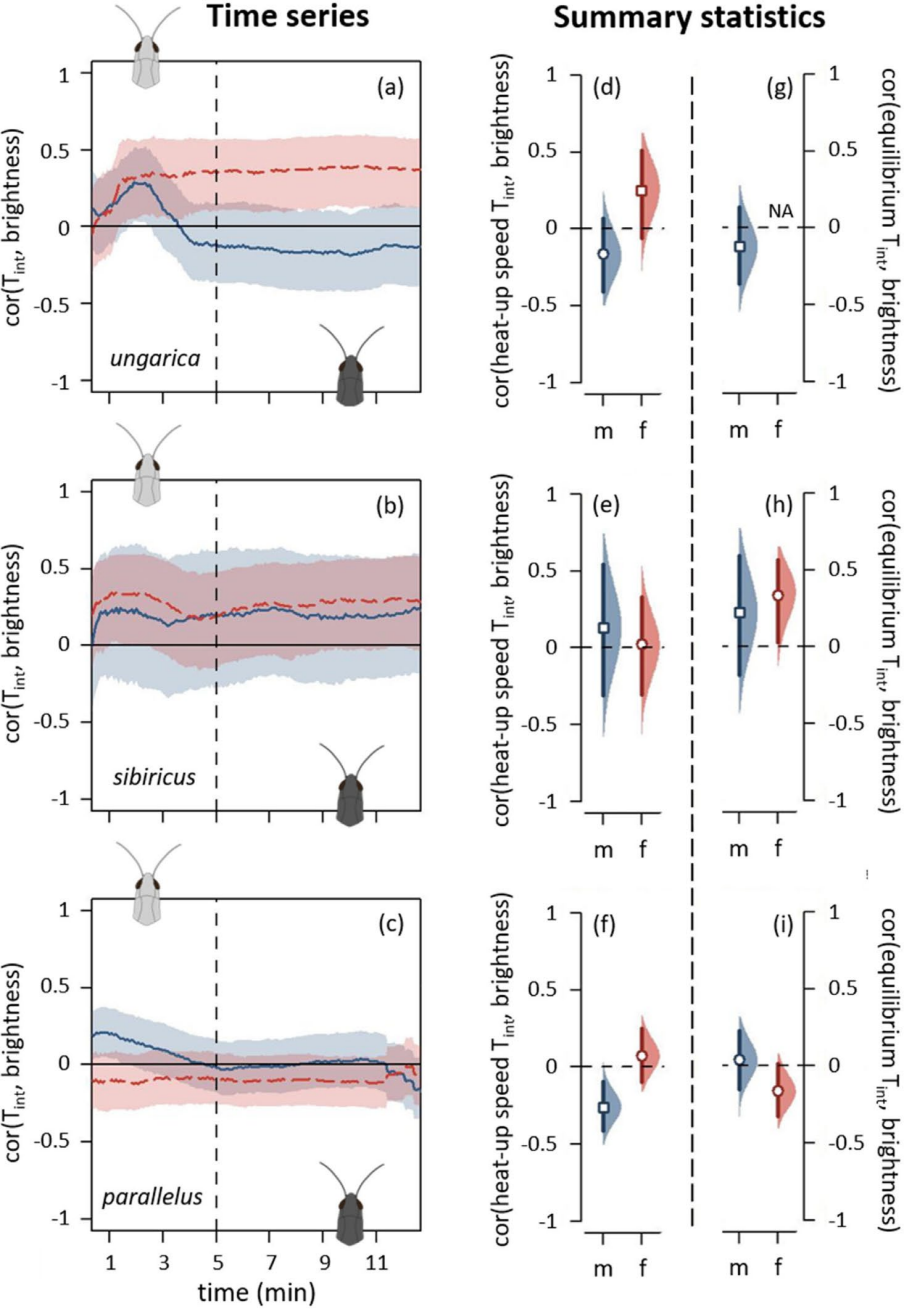
Thermal physiology depending on brightness for *Acrida ungarica*, *Gomphocerus sibiricus* and *Pseudochorthippus parallelus* (from top to bottom, abbreviated respectively *ungarica*, *sibiricus* and *parallelus*). The dotted vertical lines distinguish between the heat‐up phase (left) and the equilibrium phase (right). (a–c) Spearman's correlation coefficients between brightness and internal temperature (T_int_) depending on time. Each species' dataset was split into males (solid blue curves) and females (dashed red curves). When a curve goes above the zero line, it indicates that brighter individuals were hotter than darker individuals. See Figure 4 caption for details on graphical displays. (d–f) Correlation coefficients between internal heat‐up speed and brightness. (g–i) Correlation coefficients between internal equilibrium temperature and brightness. (d–i) Half eyes represent bootstrapped 95% confidence intervals and the corresponding bootstrap densities of the correlation coefficients. Males in blue, females in red. When a 95% confidence interval includes zero, it indicates that the correlation is not different from zero. Pearson's and Spearman's correlations are denoted by circles and squares, respectively. *Ungarica* females did not reach any equilibrium, and their equilibrium temperatures are, hence, not available (NA).

**FIGURE 7:**
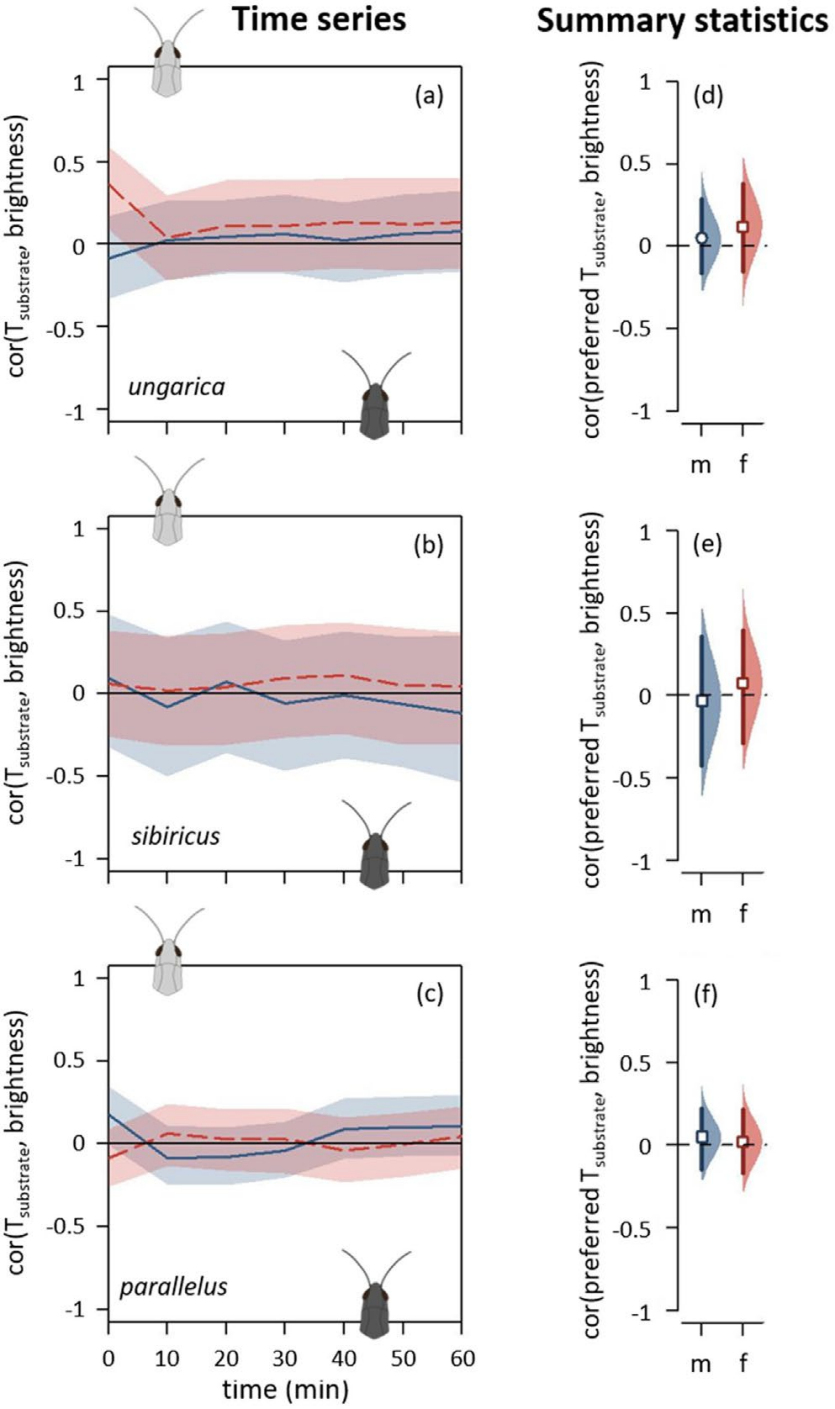
Thermal preferences depending on brightness for *Acrida ungarica*, *Gomphocerus sibiricus* and *Pseudochorthippus parallelus* (from top to bottom, abbreviated respectively *ungarica*, *sibiricus* and *parallelus*). (a–c) Spearman's correlation coefficients between brightness and substrate temperature (T_substrate_) depending on time. Each species' dataset was split into males (solid blue curves) and females (dashed red curves). When a curve goes above the zero line, it indicates that brighter individuals were standing on hotter temperatures than darker individuals. See Figure 4 caption for details on graphical displays. (d–f) Correlation coefficients between preferred temperature and brightness. See Figure 6 caption for details on graphical displays.

Brightness, when measured as colour variation independently of colour morphs, did not consistently predict the heat‐up and equilibrium phases across the three species (Figure 6, Figure S5). During the heat‐up phase, there was a tendency for brighter grasshoppers to heat up faster (Figure 6a–c, Figure S5a–c), but only in the first 2 min of the experiment (Figure 6d–f, Figure S5d–f). Brighter individuals were slightly associated with higher internal and external temperatures in *ungarica*, *parallelus* males, and a similar trend was observed for *sibiricus* (Figure 6a–c, Figure S5a–c). During the equilibrium phase, brightness had no effect on equilibrium temperature in males but contrasting effects in females, with dark *parallelus* females tending to be warmer than bright ones while dark *sibiricus* females were cooler than bright ones (Figure 6a–c,g–i, Figure S5a–c,g–i). In the thermal preference experiment, brightness did not predict preferred temperatures (Figure 7).

### 3.3 Effects of the Sex and Body Mass

Males and females differed in brightness in all three species (*ungarica*: *t* = 2.83, df = 108, *p* = 0.005; *sibiricus*: *W* = 77, *p* < 0.001; *parallelus*: *W* = 1339, *p* < 0.001). Females were darker in *ungarica* (females: 18.9% ± 3.6%, males: 20.5% ± 2.7%), but brighter in *sibiricus* and *parallelus* (*sibiricus* females: 5.1% ± 1.3%, males: 2.8% ± 1.1%; *parallelus* females: 13.2% ± 2.7%, males: 8.6% ± 2.2%). The sexes also differed in body weight (*ungarica*: *W* = 0, *p* < 0.001; *sibiricus*: *W* = 16, *p* < 0.001; *parallelus*: *W* = 23.5, *p* < 0.001) with females being heavier than males in all species (factor 5.3 in *ungarica*: females 1.12 ± 0.27 g, males 0.21 ± 0.02 g; factor 1.9 in *sibiricus*: females 0.36 ± 0.07 g, males 0.19 ± 0.03 g; factor 2.2 in *parallelus*: females 0.22 ± 0.04 g, males 0.10 ± 0.01 g). Females heated up more slowly than males (Figure 2, Figures S1 and S2a–f; Table S1). A similar pattern was found for the equilibrium temperatures. Females reached cooler equilibrium temperatures than males in *sibiricus*, but not in *parallelus* (Figure 2, Figure S1; Table S1). Females preferred cooler temperatures compared to males in *ungarica*, but not in *sibiricus* and *parallelus* (Figure S2g–i, Table S1).

**FIGURE 8:**
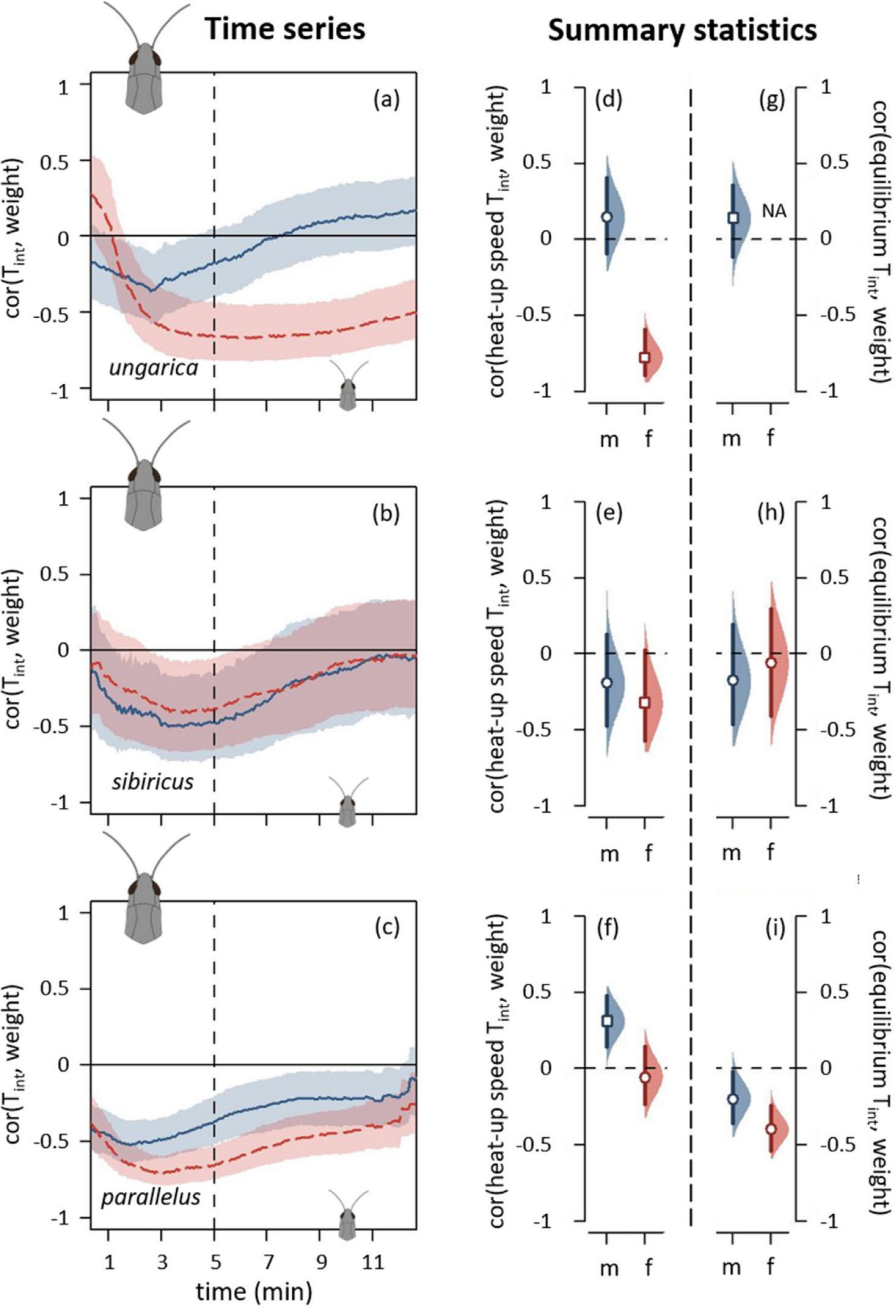
Thermal physiology depending on weight for *Acrida ungarica*, *Gomphocerus sibiricus* and *Pseudochorthippus parallelus* (from top to bottom, abbreviated, respectively, *ungarica*, *sibiricus* and *parallelus*). The dotted vertical lines distinguish between the heat‐up phase (left) and the equilibrium phase (right). (a–c) Spearman's correlation coefficients between weight and internal temperature (T_int_) depending on time. Each species' dataset was split into males (solid blue curves) and females (dashed red curves). When a curve goes above the zero line, it indicates that heavier individuals were hotter than lighter individuals. See Figure 4 caption for details on graphical displays. (d–f) Correlation coefficients between internal heat‐up speed and weight. (g–i) Correlation coefficients between internal equilibrium temperature and weight. (d–i) See Figure 6 caption for details on graphical displays. *Ungarica* females did not reach any equilibrium, and their equilibrium temperatures are, hence, not available (NA).

**FIGURE 9:**
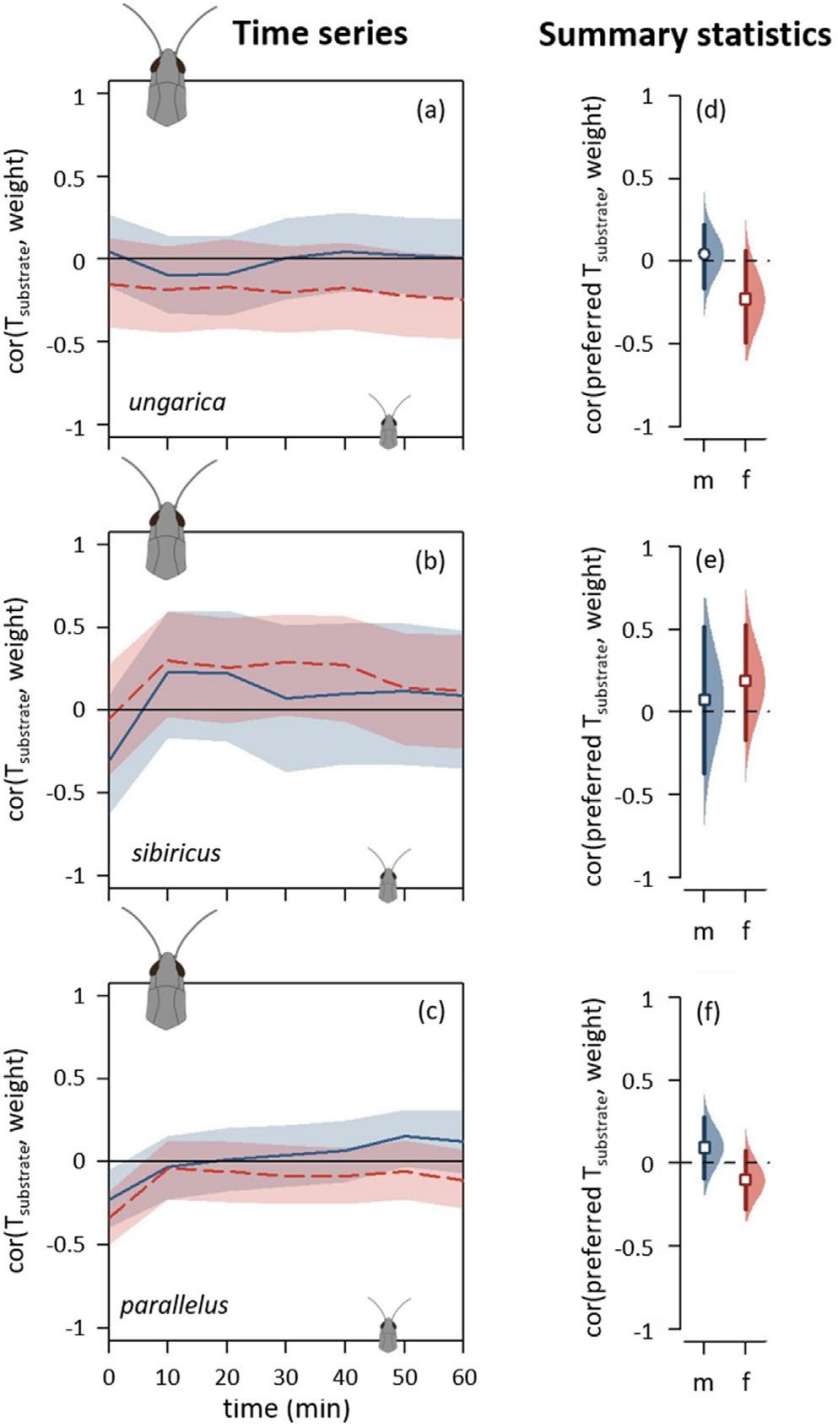
Thermal preferences depending on weight for *Acrida ungarica*, *Gomphocerus sibiricus* and *Pseudochorthippus parallelus* (from top to bottom, abbreviated, respectively, *ungarica*, *sibiricus* and *parallelus*). (a–c) Spearman's correlation coefficients between weight and substrate temperature (T_substrate_) depending on time. Each species' dataset was split into males (solid blue curves) and females (dashed red curves). When a curve goes above the zero line, it indicates that heavier individuals were standing on hotter temperatures than lighter individuals. See Figure 4 caption for details on graphical displays. (d–f) Correlation coefficients between preferred temperature and weight. See Figure 6 caption for details on graphical displays.

Body weight shaped the heat‐up phase in a similar way across all three species: Heavier grasshoppers heated more slowly (Figure 8a–c, Figure S6a–c). The analyses on the heat‐up speed confirmed this pattern, though less consistent across species with a markedly stronger effect in females than in males (Figure 8d–f, Figure S6d–f). This pattern faded away during the course of the heat‐up phase until no difference was detected towards the end of the equilibrium phase (Figure 8a–c, Figure S6a–c). Only *parallelus* retained this difference when considering the equilibrium temperature (Figure 8c,g–i, Figure S6c,g–i). Grasshoppers of different body weights had similar preferred temperatures, with the potential exception of *ungarica* females, for which larger females tended to prefer cooler temperatures (Figure 9).

## 4 Discussion

We analysed the thermal physiology and temperature preferences of three species of green–brown polymorphic grasshoppers. Since brown individuals were hotter than green conspecifics (Cheng et al. 2022; Köhler and Schielzeth 2020), we expected the brown morph to heat up faster and to reach higher equilibrium temperatures than the green morph. Our results demonstrate that they do not differ in their heat‐up or equilibrium patterns. Morph‐specific body temperature differences in the field are therefore not the outcome of thermal physiology, but might instead result from morph‐specific behavioural preferences. Our results show that colour morphs did not differ in thermal preferences. Hence, field observations would likely be the outcome of behavioural preferences for non‐thermal requirements that happen to correlate with thermal parameters. Additionally, our results demonstrate that the green–brown polymorphism is not following the thermal melanism hypothesis since darker individuals did not heat up faster nor did they reach higher equilibrium temperatures than brighter individuals. Interestingly, males heated up more quickly and tended to reach higher equilibrium temperatures and to prefer higher temperatures. However, this effect might be (directly or indirectly) mediated by body size since the strongest associations occurred with body mass. Notably, the conclusions based on invasive and non‐invasive methods (internal and external temperatures, respectively) were overall similar, but when differences were detected, the statistical power was greater with the invasive method.

### 4.1 Discrete Colour Morphs

Field studies on green–brown polymorphic grasshoppers have shown that different colour morphs differ in body temperatures, including two of the species that we studied here. These previous studies revealed that brown individuals were on average warmer than their green conspecifics by 1.2°C in *Oedaleus decorus*, and 1.5°C in *Gomphocerus sibiricus* and *Pseudochorthippus parallelus* (Cheng et al. 2022; Köhler and Schielzeth 2020). These findings reflect the effects of thermal physiology and/or behavioural thermoregulation. Unlike field studies, our experimental set‐up allowed us to test specifically for the thermal physiology of colour morphs. We showed that green and brown individuals of three different species had similar heat‐up patterns and similar equilibrium temperatures. Hence, the thermoregulatory properties of body colour are unlikely to explain the field results. Some other studies found no effect of colour morphs on thermal physiology in the two‐spot ladybird and also in other grasshopper species (Cheng et al. 2022; Umbers et al. 2013; de Jong et al. 1996; Stower and Griffiths 1966). This leaves behavioural thermoregulation and background colour matching as alternative explanations for body temperature differences in the field.

Behavioural thermoregulation can be achieved by choosing microniches with specific thermal characteristics (Karpestam et al. 2012; Wennersten et al. 2012; Forsman et al. 2002; Whitman 1987). Since we found no differences in thermal preferences between colour morphs, we reject that field results can be explained by the choice of a microhabitat based on its thermal characteristics. Nonetheless, microniche choice might occur for reasons other than thermal properties, such as crypsis, and still have thermal consequences. To achieve crypsis, brown individuals should preferentially choose bare soil and dry grass patches, while green individuals should choose fresh grass patches—that is, performing background colour matching. Bare soil patches have been found to be hotter than grassy patches inhabited by *sibiricus* (Cosandey et al. 2021). If grasshoppers are selecting microhabitat for crypsis, background colour matching could thus explain the differences in body temperature observed in the field. Alternatively, behavioural thermoregulation can be achieved by adopting specific positions such as basking or stilting (Whitman 1987). If this feature of behavioural thermoregulation is linked to colour morphs, field results could be explained. There is evidence for such linkage in the groundhoppers *Tetrix subulata* , where brown individuals bask for longer periods than green conspecifics (Forsman et al. 2002). The green–brown polymorphism in acridid grasshoppers is known to be genetically based for *Gomphocerus sibiricus*, *Pseudochorthippus parallelus, Chorthippus dorsatus* and probably other Gomphocerinae (Winter et al. 2021; Schielzeth and Dieker 2020; unpublished own data). A linkage could thus have a genetic basis by pleiotropy, cascading effects or physical linkage of genes underlying behaviour and colour variation. The mechanisms could be different in the colour‐changing *Acrida ungarica* since it is unknown for this species if there is a genetic component to colour variation and if morphs have different body temperatures in the field.

Radiant heat gain has been shown to contribute up to 50% of the total heat gain in the stripe‐winged grasshopper *Stenobothrus lineatus* (Samietz and Köhler 2020). In our laboratory setting, the amount of radiant heat was much less than under sunny field conditions. However, we had expected that any difference would be most pronounced under limiting conditions when heat‐up is challenging. This condition would also be ecologically relevant since many days are not fully sunny and heat gain under limiting conditions seems particularly important for grasshoppers. We thus consider our experimental setting suitable for capturing the critical aspect of the thermal ecology of colour variants. The fact that there was no effect of either thermal physiology or thermal preferences was initially surprising. Although it provides important information for understanding the maintenance of green–brown polymorphism, since it does not seem to be the thermal properties of the colour morphs that contribute to a balanced maintenance of colour variation.

### 4.2 Continuous Colour Variation

The thermal melanism hypothesis predicts that darker individuals would have a thermal advantage over brighter individuals at colder conditions, while brighter individuals would be advantaged in warmer conditions (Zeuss et al. 2014). We counterintuitively found a tendency for brighter grasshoppers to heat up faster, though this pattern was not consistent across species and sexes. Contrastingly, it has been shown for melanic polymorphic grasshoppers and ladybirds that the blackish morph reached a body temperature higher than the non‐melanic morph (Harris et al. 2013; Forsman et al. 2002; Forsman 1997; de Jong et al. 1996). The blackish ladybirds reflected significantly less light compared to non‐melanic conspecifics (Brakefield and Willmer 1985), and considering the striking difference in *Tetrix* morphs (see pictures here: Johansson et al. 2013), it would likely bring similar results if reflectance had been measured. However, in the chameleon grasshopper *Kosciuscola tristis* (i.e., able to switch between colour states within hours), the black state reflected less light than the turquoise state, but did not heat up faster nor did it reach higher body temperatures (Umbers et al. 2013). While the thermal melanism hypothesis seems reliable interspecifically (Zeuss et al. 2014; Clusella‐Trullas et al. 2008; Rapoport 1969), it seems to have poor explanatory power for grasshopper species. This is likely because, when reaching sufficient body temperatures is not limiting, discrete as well as continuous colour variation may have evolved for different functions. It could have evolved for other colour‐relevant functions such as crypsis or even for colour‐irrelevant functions such as immunity since melanin plays a role in immunity in ectotherms (crypsis: Franks and Oxford 2011; immunity: Mikkola and Rantala 2010; Civantos et al. 2005). Alternatively, it could have evolved for no function at all if correlated with other traits (McKinnon and Pierotti 2010; True 2003).

### 4.3 Sex and Body Mass

Larger individuals have a smaller surface‐to‐volume ratio, implying a greater thermal inertia (Stevenson 1985). Our grasshoppers were no exception to the rule, as heavier individuals heated up more slowly. Female grasshoppers were systematically heavier than their conspecific males, heated up more slowly and attained cooler equilibrium temperatures in *sibiricus*. We think that sex differences are largely mediated by size differences. *Ungarica* females did not reach any equilibrium within the time frame of the experiment, likely due to their markedly greater thermal inertia (5.3, 3.1 and 5.1 times heavier than *ungarica* males, *parallelus* females and *sibiricus* females, respectively).

Even though sex differences can largely be attributed to the overall differences in body mass, they can still be of ecological relevance since they can impose different thermal constraints on each sex. First, they could mediate the effect of natural selection on the two sexes. Forsman (1999a) showed that grasshoppers are better at escaping predators at higher body temperatures. Males might thus be better at escaping predation than females, leading to a stronger selective pressure imposed by predators on female grasshoppers. *Ungarica* males preferred higher temperatures than their conspecific females by about 2.8°C. This suggests that thermal physiology and behaviour evolved in concert in *ungarica* females, possibly to reduce the potential costs inherent to a greater body mass. Second, the sex difference could have implications on egg production in females. Insect females produce fewer eggs per unit time when maintained at cooler temperatures (Francuski et al. 2020; Brent and Spurgeon 2019; Berger et al. 2008). Larger grasshopper females would be expected to produce fewer eggs during bouts of cool days, that is, when weather conditions are limiting. Hence, the fitness of females might be the outcome of the interaction between weather conditions and body mass, since larger insects produce more eggs (Marshall et al. 2013; Berger et al. 2008; Forsman 1999b). In males, however, sperm production was found to be independent of temperature or reduced only by extreme temperatures in insect species from various orders (Malawey et al. 2021; Vasudeva et al. 2014; Stürup et al. 2013). Hence, the fitness of differently sized males would mostly result from their longevity and mating success, and little if none on body mass‐dependent heating.

## 5 Conclusion

The green–brown polymorphism, which is widely shared in orthopterans, appears to be a long‐term maintained polymorphism whose maintenance mechanism is currently unknown (Schielzeth 2021). This polymorphism could have evolved to meet thermal requirements, as suggested by field results on the body temperature of green and brown grasshoppers (Cheng et al. 2022; Köhler and Schielzeth 2020), for which the thermal melanism hypothesis would be a mechanistic explanation. Our results, however, do not support the thermal melanism hypothesis for the green–brown polymorphism, likely because between‐morph brightness differences are overall small and because brightness as such does not influence thermal physiology in these species. Hence, field results were not mediated by thermal physiology. Alternatively, the body temperature of green and brown grasshoppers could have been the outcome of temperature preferences, but our results do not support this hypothesis either.

These findings are important for understanding the maintenance of the colour polymorphism in Orthoptera. The long‐term maintenance of the green–brown polymorphism may result from other mechanisms such as niche partitioning via microhabitat choice (Whitney et al. 2018), mating preferences (Pryke and Griffith 2007) or negative‐frequency‐dependent apostatic selection (Bond 2007). Furthermore, we showed a rather strong effect of body weight in shaping heat‐up patterns but not equilibrium temperatures, reaffirming the importance of body mass in the thermal physiology of ectotherms. Our results also suggest changes in temperature and radiation regimes will not affect colour morph composition via thermal physiology, but it might favour larger species (and individuals) that heat up more slowly under limiting conditions.

## Author Contributions

**Lilian Cabon:** conceptualization (equal), data curation (equal), formal analysis (lead), investigation (lead), visualization (lead), writing – original draft (lead), writing – review and editing (equal). **Holger Schielzeth:** conceptualization (equal), data curation (equal), formal analysis (supporting), funding acquisition (lead), methodology (lead), resources (lead), supervision (lead), writing – review and editing (equal).

## Conflicts of Interest

The authors declare no conflicts of interest.

## Supporting information


Data S1.

## Data Availability

Data and code are available at https://doi.org/10.5061/dryad.cc2fqz6g3.
