# Supplementary material for: Body Temperature Differences Between Green And Brown Grasshoppers Do Not Result From Thermal Physiology or Thermal Preferences
Source: Ecol Evol. 2025 Mar 11;15(3):e71104. doi: 10.1002/ece3.71104 (PMC11896881; doi:10.1002/ece3.71104)
Supplement: Supplementary file 1 — Data S1. [file ECE3-15-e71104-s001.docx]

# Supplementary materials

**Figure S1**: External temperature depending on time for both sexes (solid blue curves: males, dashed red curves: females) of *Acrida ungarica*, *Gomphocerus sibiricus*, and *Pseudochorthippus parallelus* (left to right). Curves represent the average heat-up trajectories; ribbons represent the standard deviations.


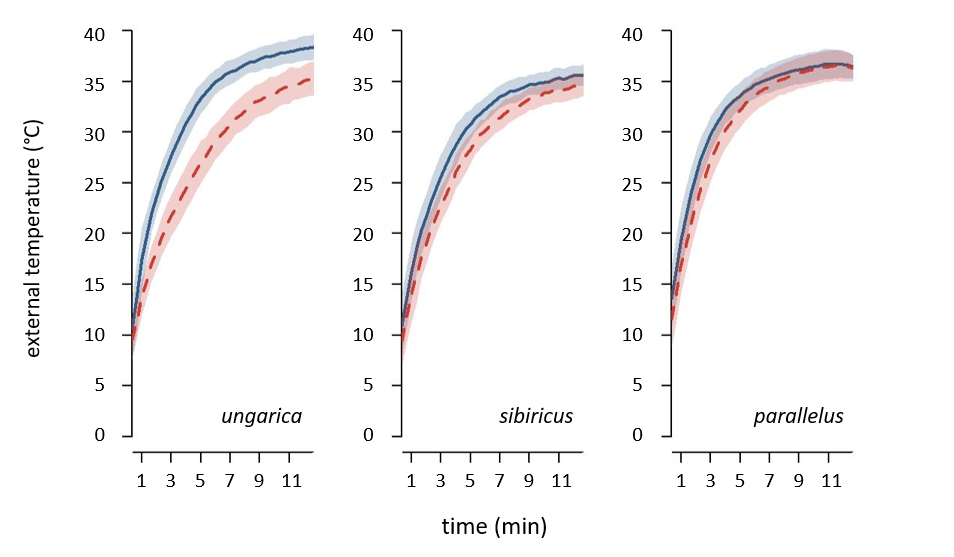


**Figure S2**: Individual trajectories for internal (**a-c**), external (**d-f**) and substrate (**g-i**) temperatures depending on time. Males are plotted in blue, and females in red. *Acrida ungarica* (**a**, **d**, **g**), *Gomphocerus sibiricus* (**b**, **e**, **h**), and *Pseudochorthippus parallelus* (**c**, **f**, **i**).


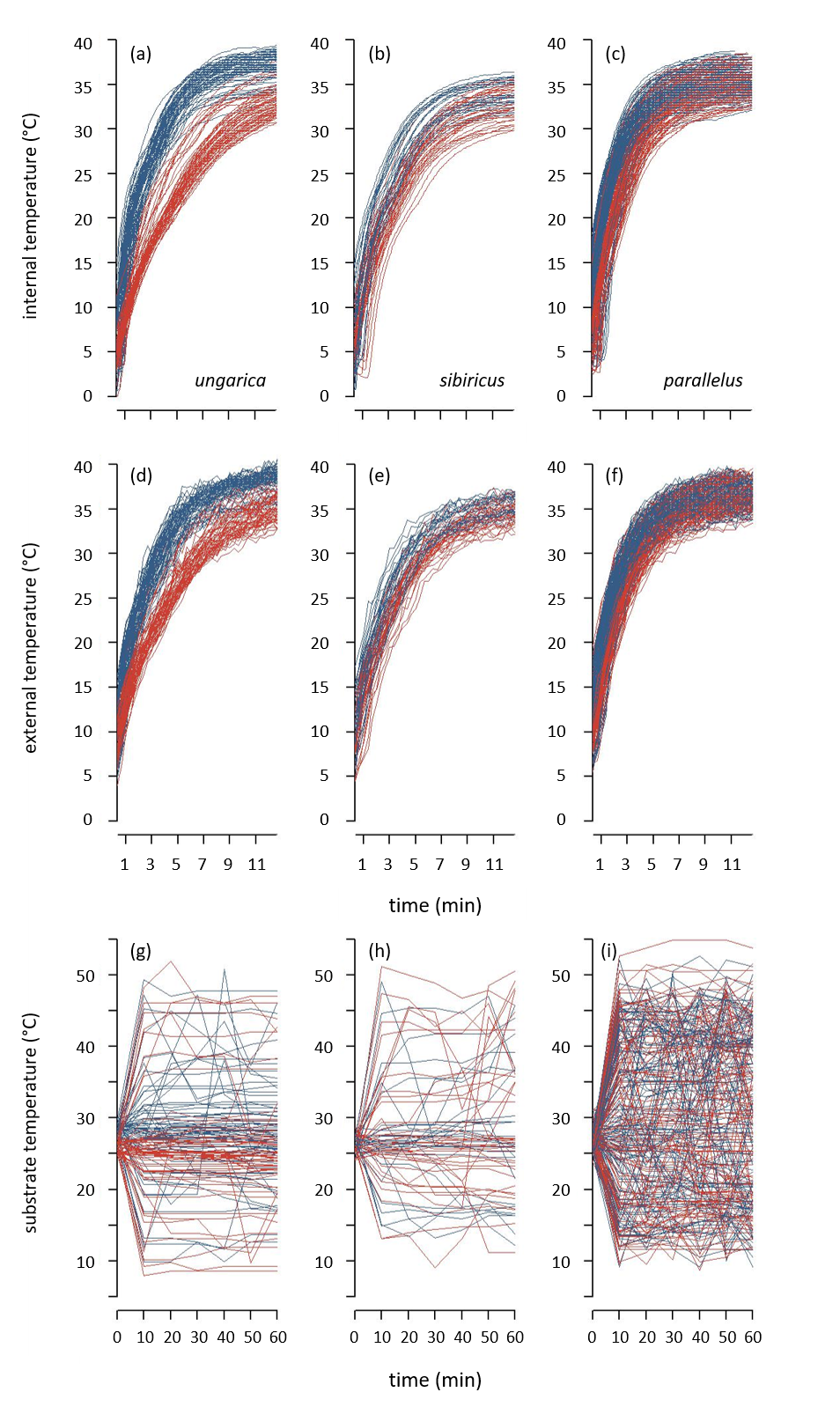


**Figure S3: Thermal physiology depending on morphs** for *Acrida ungarica* (**top**), *Gomphocerus sibiricus* (**middle**), and *Pseudochorthippus parallelus** (**bottom**). The dotted vertical lines distinguish between the heat-up phase (left) and the equilibrium phase (right). **a-c**: Pairwise differences in external temperature (T_ext_) between green and brown morphs depending on time (T_green_ – T_brown_). Each species’ dataset was split into males (solid blue curves) and females (dashed red curves). When a curve goes above the zero line, it indicates that the green morph was hotter than the brown morph. Curves represent average differences. Ribbons represent bootstrapped 95% confidence intervals. The dashed vertical lines separate the heat-up phase (before) and the equilibrium phase (after). **d-f**: external heat-up speed. **g-i**: external equilibrium temperature. **d-i**: Violins represent densities of observations and their boxplots with medians indicated by a white dot. G, D, L, and B stand for green, dorsal-green, lateral-green, and brown morphs, and are shown in green, grey, grey, and brown, respectively. NS means non-significant. *Ungarica* females did not reach any equilibrium, and their equilibrium temperatures are, hence, not available (NA). *For the pairwise differences involving the two intermediate morphs (dorsal green and lateral green), see Figure S4f-j in supplementary materials.


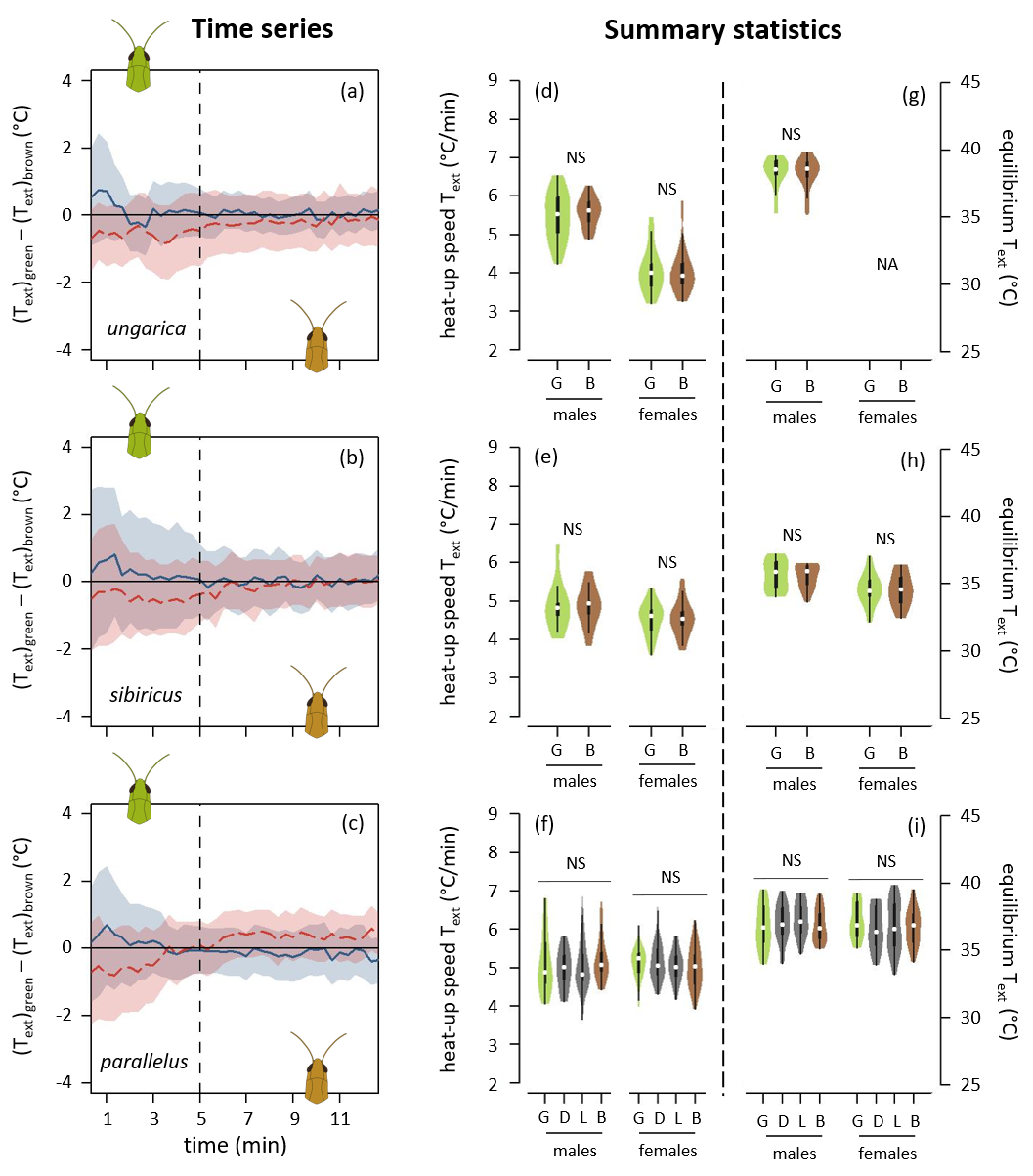


**Figure S4**: Morph pairwise differences (ΔT) involving either/both intermediate morphs (dorsal green and lateral green) depending on time for *Pseudochorthippus parallelus*. The dataset for *parallelus* was split into males (solid blue curves) and females (dashed red curves). **a-e:** internal temperature (T_int_) differences from the heat-up experiment.
**f-i:** external temperature (T_ext_) differences from the heat-up experiment. **a-j**: When a curve goes above the zero line, it indicates that the reference morph was hotter than the morph that was subtracted from it. The dotted vertical lines distinguish between the heat-up phase (left) and the equilibrium phase (right). **k-o:** substrate temperature (T_substrate_) differences from the thermal gradient experiment. When a curve goes above the zero line, it indicates that the reference morph was standing on hotter temperatures than the morph that was subtracted from it. Curves represent the average differences; ribbons represent the bootstrapped 95% confidence intervals.


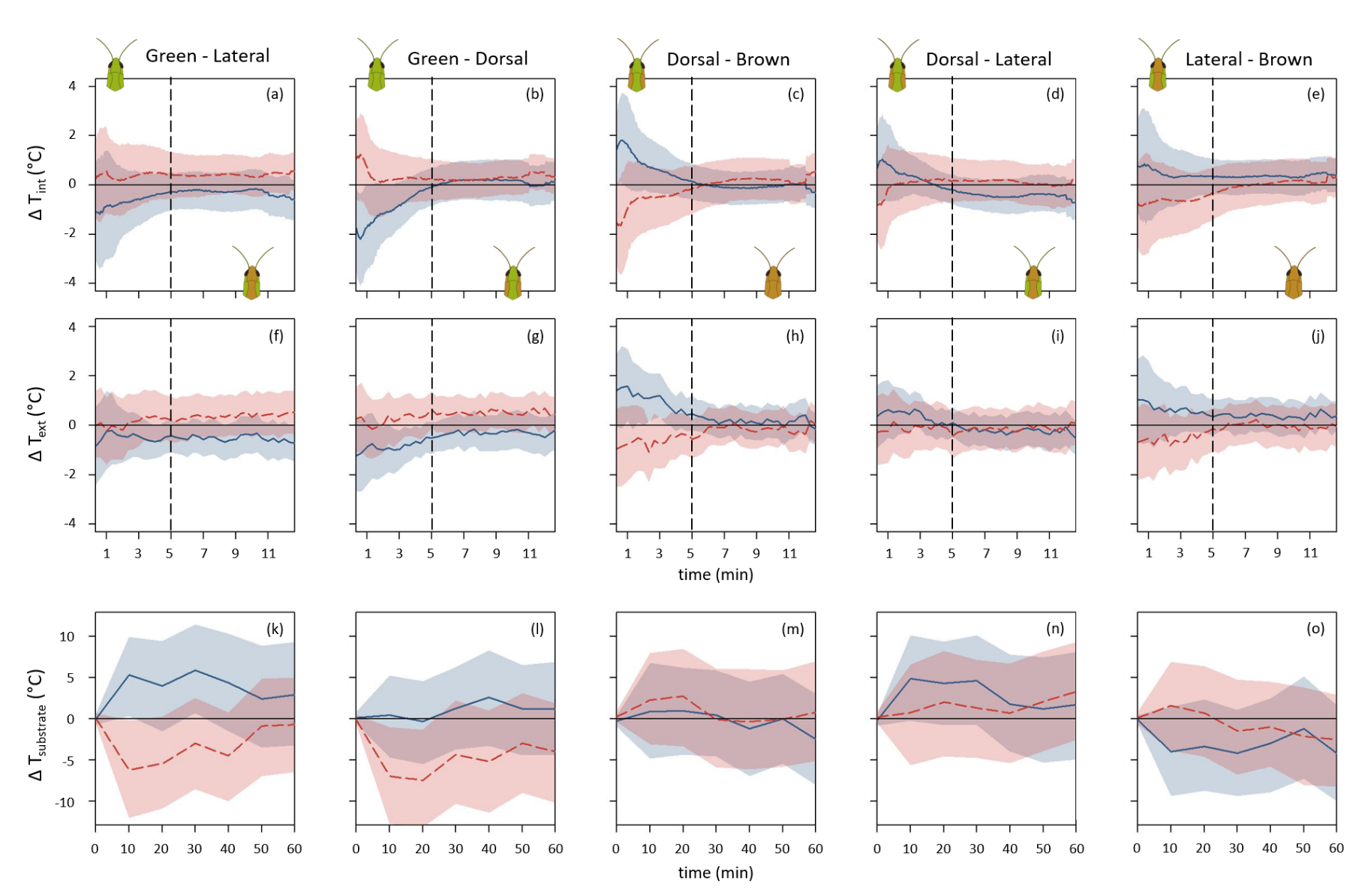


**Figure S5: Thermal physiology depending on brightness** for *Acrida ungarica* (**top**), *Gomphocerus sibiricus* (**middle**), and *Pseudochorthippus parallelus* (**bottom**). The dotted vertical lines distinguish between the heat-up phase (left) and the equilibrium phase (right). **a-c**: Spearman’s correlation coefficients between brightness and external temperature (T_ext_) depending on time. Each species’ dataset was split into males (solid blue curves) and females (dashed red curves). When a curve goes above the zero line, it indicates that brighter individuals were hotter than darker individuals. See figure S4 caption for details on graphical displays. **d-f**: Correlation coefficients between external heat up speed and brightness. **g-i**: Correlation coefficients between external equilibrium temperature and brightness. **d-i**: Halfeyes represent bootstrapped 95% confidence intervals and the corresponding bootstrap densities of the correlation coefficients. Males in blue, females in red. When a 95% confidence interval includes zero, it indicates that the correlation is not different from zero. Pearson’s and Spearman’s correlations are denoted by circles and squares, respectively. *Ungarica* females did not reach any equilibrium, and their equilibrium temperatures are, hence, not available (NA).

**
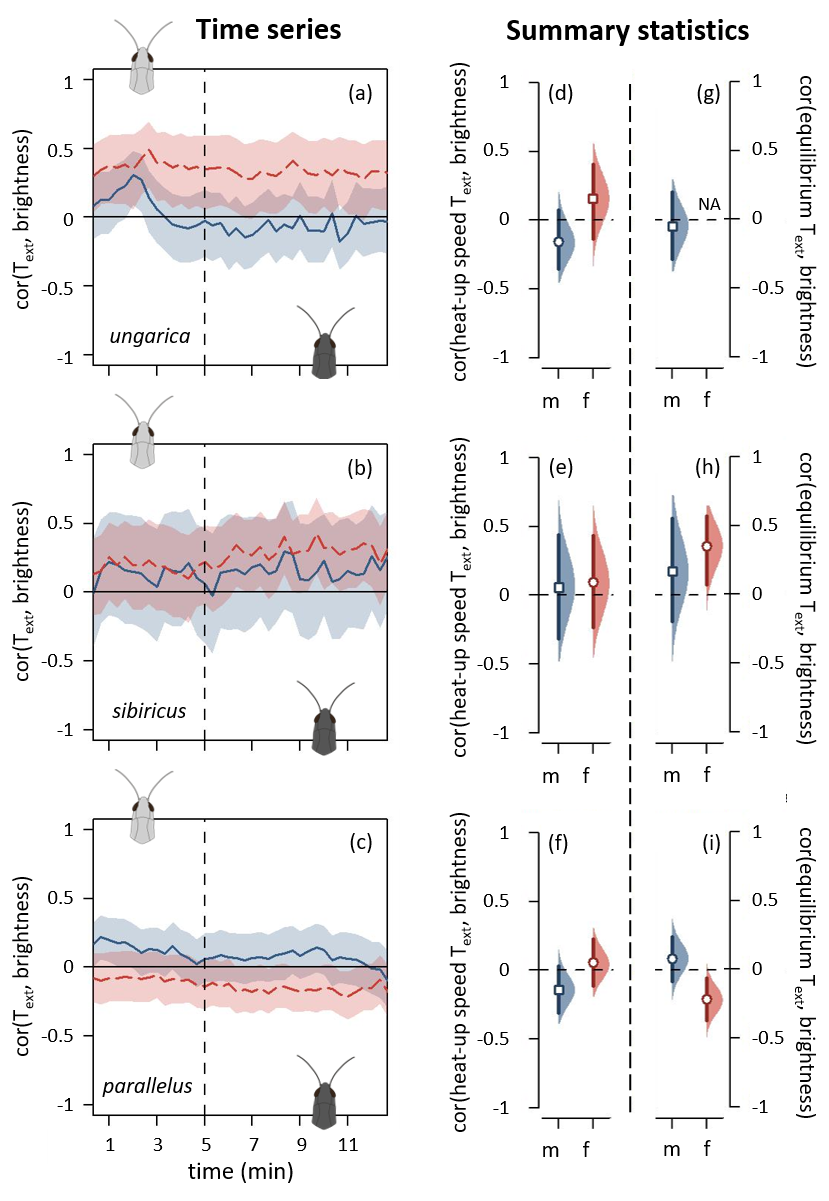
**

**Figure S6: Thermal physiology depending on weight** for *Acrida ungarica* (**top**), *Gomphocerus sibiricus* (**middle**), and *Pseudochorthippus parallelus* (**bottom**). The dotted vertical lines distinguish between the heat-up phase (left) and the equilibrium phase (right). **a-c**: Spearman’s correlation coefficients between weight and external temperature (T_ext_) depending on time. Each species’ dataset was split into males (solid blue curves) and females (dashed red curves). When a curve goes above the zero line, it indicates that heavier individuals were hotter than lighter individuals. See figure S4 caption for details on graphical displays. **d-f**: Correlation coefficients between external heat-up speed and weight. **g-i**: Correlation coefficients between external equilibrium temperature and weight. **d-i**: See figure S5 caption for details on graphical displays. *Ungarica* females did not reach any equilibrium, and their equilibrium temperatures are, hence, not available (NA).


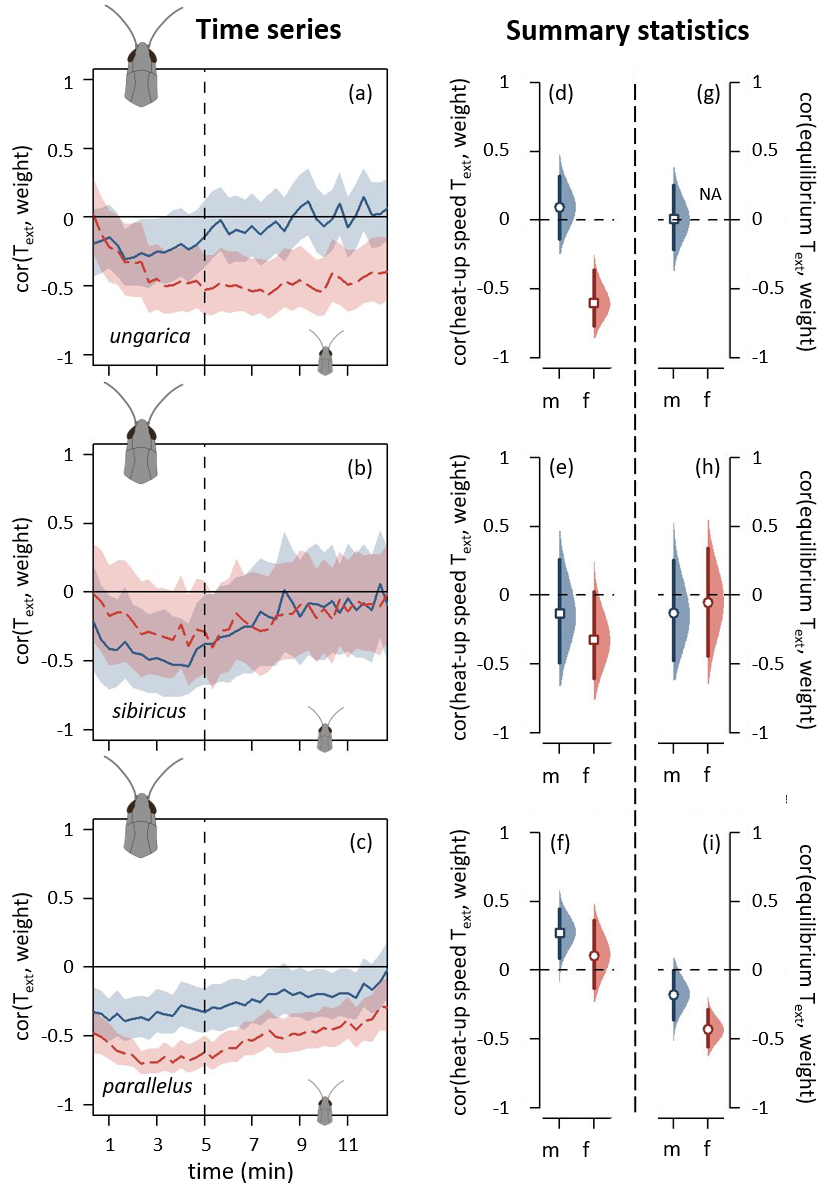


Table S1: Sex differences in heat-up speeds, equilibrium temperatures, and preferred temperatures of *Acrida ungarica*, *Gomphocerus sibiricus*, and *Pseudochorthippus parallelus*. Heat-up speeds and equilibrium temperatures come from the heat-up experiment; preferred temperature comes from the thermal gradient experiment. Comparison means tests are Mann-Whitney tests or t tests (* shows when t tests have been performed; statistics are t (df), and *p*). Tests on equilibrium temperature of *ungarica* females have not been performed because no equilibrium was reached. Bold p-values indicate tests with a significance level below 0.05.

|  |  | *Acrida ungarica* | | | | | *Gomphocerus sibiricus* | | | |  | *Pseudochorthippus parallelus* | | | |
| --- | --- | --- | --- | --- | --- | --- | --- | --- | --- | --- | --- | --- | --- | --- | --- |
| **Heat-up speed (°C.min^-1^)** | | | | | | | | | | | | | | | |
|  |  | W | *p* | females | males |  | W | *p* | females | males |  | W | *p* | females | males |
|  | internal temperature | 3836 | **<0.001** | 4.3 ± 0.7 | 6.3 ± 0.6 |  | *4.72 (62) | **<0.001** | 4.9 ± 0.5 | 5.6 ± 0.8 |  | 6029 | **0.04** | 5.5 ± 0.6 | 5.4 ± 0.7 |
|  | external temperature | 3878 | **<0.001** | 4.1 ± 0.6 | 5.5 ± 0.5 |  | 671 | **0.02** | 4.5 ± 0.4 | 4.9 ± 0.6 |  | 6416 | 0.35 | 5.1 ± 0.5 | 5.1 ± 0.6 |
| **Equilibrium temperature (°C)** | | | | | | | | | | | | | | | |
|  |  |  |  | females | males |  | W | *p* | females | males |  | W | *p* | females | males |
|  | internal temperature | NA | | NA | 37.1 ± 1.4 |  | *3.73 (62) | **<0.001** | 32.7 ± 1.7 | 34.2 ± 1.4 |  | *0.65 (233) | 0.52 | 35.3 ± 1.5 | 35.4 ± 1.5 |
|  | external temperature | NA | | NA | 38.2 ± 1.1 |  | 737 | **<0.001** | 34.4 ± 1.2 | 35.5 ± 1.0 |  | 7331 | 0.43 | 36.7 ± 1.5 | 36.8 ± 1.5 |
| **Preferred temperature (°C)** | | | | | | | | | | | | | | | |
|  |  | W | *p* | females | males |  | W | *p* | females | males |  | W | *p* | females | males |
|  |  | 2676 | **<0.01** | 25.6 ± 8.2 | 28.4 ± 6.7 |  | 480 | 0.85 | 28.5 ± 8.4 | 27.4 ± 8.3 |  | 7590 | 0.40 | 29.6 ± 10.7 | 30.8 ± 10.0 |
